# Tumorigenic Cell Reprogramming and Cancer Plasticity: Interplay between Signaling, Microenvironment, and Epigenetics

**DOI:** 10.1155/2018/4598195

**Published:** 2018-05-02

**Authors:** Vittoria Poli, Luca Fagnocchi, Alessio Zippo

**Affiliations:** ^1^Laboratory of Chromatin Biology and Epigenetics, Center for Integrative Biology (CIBIO), University of Trento, 38123 Trento, Italy; ^2^Department of Epigenetics, Fondazione Istituto Nazionale di Genetica Molecolare “Romeo ed Enrica Invernizzi”, Via F. Sforza 35, 20122 Milan, Italy; ^3^Division of Pathology, Fondazione IRCCS Ca' Granda Ospedale Maggiore Policlinico, Milan, Italy

## Abstract

Accumulating evidences indicate that many tumors rely on subpopulations of cancer stem cells (CSCs) with the ability to propagate malignant clones indefinitely and to produce an overt cancer. Of importance, CSCs seem to be more resistant to the conventional cytotoxic treatments, driving tumor growth and contributing to relapse. CSCs can originate from normal committed cells which undergo tumor-reprogramming processes and reacquire a stem cell-like phenotype. Increasing evidences also show how tumor homeostasis and progression strongly rely on the capacity of nontumorigenic cancer cells to dedifferentiate to CSCs. Both tumor microenvironment and epigenetic reprogramming drive such dynamic mechanisms, favoring cancer cell plasticity and tumor heterogeneity. Here, we report new developments which led to an advancement in the CSC field, elucidating the concepts of cancer cell of origin and CSC plasticity in solid tumor initiation and maintenance. We further discuss the main signaling pathways which, under the influence of extrinsic environmental factors, play a critical role in the formation and maintenance of CSCs. Moreover, we propose a review of the main epigenetic mechanisms whose deregulation can favor the onset of CSC features both in tumor initiation and tumor maintenance. Finally, we provide an update of the main strategies that could be applied to target CSCs and cancer cell plasticity.

## 1. Introduction

Cancer is a heterogeneous group of diseases caused by genetic and epigenetic changes conferring key properties to cancer cells, including chronic proliferation, resistance to cell death, replicative immortality, invasiveness, and metastatic potential. In addition, interactions between tumor cells and the microenvironment are a crucial determinant of malignant growth [1]. Almost all human tumors are characterized by a considerable intratumor heterogeneity, with cancer cells showing different phenotypes, gene expression patterns, and proliferation potentials. Moreover, different patients affected by the same cancer type show a significant intertumor heterogeneity. Intra- and intertumor heterogeneity mostly account for difficulties in the development of effective therapies and new targeted agents [2].

Among the factors that have been proposed to explain intra- and intertumor heterogeneity and therapy resistance, a critical aspect is represented by the different potential shown by cancer cells in driving tumorigenesis and cancer progression. Specifically, the uncontrolled growth of many tumors is driven by a population of cancer cells, known as cancer stem cells (CSCs), endowed with self-renewing and differentiation capacity. Unlike bulk cancer cells, CSCs are able to generate an overt cancer and propagate malignant clones indefinitely [3]. It follows that, at least in the early stages of tumor development, most cancers are characterized by a hierarchical organization, similar to that of healthy tissues, in which CSCs stand at the top of the hierarchy and give rise to more differentiated cancer cells. Intratumor heterogeneity can be mainly explained by different grades of differentiation between CSCs and their progeny.

It is important to note that the CSC does not necessarily coincide with the cell of origin (CO), namely, the nonneoplastic cell which acquires the first oncogenic hit [4]. Notably, intertumor heterogeneity can be the consequence of two main mechanisms: in one case, a certain CO can be affected by different combinations of genetic and epigenetic aberrations; alternatively, different cell types within the same tissue can serve as CO [4]. In both situations, cell transformation will generate CSCs with different phenotypes, which will give rise to different tumor subtypes.

Increasing evidences indicate that CSCs may originate from transformation of adult stem cells (SCs) as well as from committed progenitor cells. In the case in which cell transformation affects a committed progenitor, such CO has to undergo a dedifferentiation process in which it will lose its identity and will reacquire SC features, in order to evolve in a CSC. As a consequence, the phenotype of the CO will consistently differ from that of the corresponding CSC. It is important to note that these mechanisms not only are exclusive of the tumor initiation phase but can also take place in differentiated cancer cells in the overt tumor. Specifically, it has been shown that, during tumor progression, nonstem cancer cells undergo cell reprogramming processes and reenter the CSC state [5]. In this regard, it is becoming increasingly evident that not all cancers show a fixed hierarchical organization but can be characterized by cell plasticity, a condition in which the pool of CSCs is continuously regenerated and changes its features during tumor progression.

The aim of this review is at discussing the recent findings on the concepts of CSC and CO and describing how cell reprogramming processes play a critical role both at a pretumoral state and in tumor homeostasis and progression. We will focus on the molecular pathways and epigenetic mechanisms regulating CSC function and self-renewing, whose deregulation in a normal cell, or in a nonstem cancer cell, can drive CSC formation. In this regard, we will provide new insights in the concept of cancer cell plasticity, describing the reversible epigenetic states which control cell identity and differentiation state. Thereafter, we will elucidate how the cellular and molecular mechanisms underlying these processes are involved in clinical phenomena such as the recurrence of many tumors, after initially successful medical therapies. Finally, we will explore possible strategies that could be applied to improve the efficacy of therapies targeting signaling pathways driving CSC maintenance and cancer cell plasticity.

## 2. Cancer Stem Cells and Their Cell of Origin

The existence of CSCs was first proven in the context of acute myeloid leukemia (AML), where surface markers were used to distinguish transplantable subpopulation of malignant cells with tumor-initiating ability, from the remaining AML cells [6, 7]. Ten years later, CSCs were isolated also in solid tumors, in particular in breast carcinomas and glioblastoma (GBM), using appropriate cell surface markers [8, 9]. Intratumor heterogeneity and intertumor heterogeneity represent a consistent challenge for the identification of the CO from which CSCs derive. Moreover, cancer is an evolving entity; therefore, it is improbable that the CSC subpopulation of an advanced tumor maintained the initial phenotypic and molecular characteristics that could trace back to the CO [4].

CSCs share several features with adult SCs: they can both self-renew, forming identical daughter cells, and differentiate into different types of progenitor cells [10]. Notably, whereas adult SCs self-renew in a highly regulated manner, CSCs do so in a poorly controlled way, and while SCs generate functional mature cells, CSCs often differentiate abnormally. The SC programs in normal and cancer cells rely on many common molecular regulators. Moreover, adult SCs are relatively long lived and therefore able to accumulate mutations over time. Together, these considerations have reasonably led to deem adult SCs as the initial targets of oncogenic transformation [11]. This is well exemplified in colorectal cancer in which it has been demonstrated that CSCs originate from the tissue-specific SCs. In the small intestine epithelium, LGR5^+^ SCs are located in the crypt bottoms and are responsible for tissue homeostasis [12]. LGR5^+^ SCs are intermingled with Paneth cells, which provide instructive signals within the niche. The upper part of the crypt is occupied by transit-amplifying (TA) cells, which give rise to mature cells with a secretory or absorptive activity, located at the summit of the villus. The study of Barker et al. demonstrates that in intestinal cancer, a hierarchical organization reminiscent of the normal tissue is maintained [13, 14]. Indeed, after a single oncogenic hit, represented by mutation of the tumor suppressor APC, only LGR5^+^ cells are able to generate an overt cancer, while the same lesion occurring in TA cells is not sufficient to induce neoplasia. Nevertheless, there are observations which must be taken into account that impede to generalize this conclusion. Indeed, a series of recent studies showed that tissue hierarchies are not necessarily static but can be highly plastic, with committed progenitor cells able to reenter in the SC niche and reacquire SC features, under certain conditions. Specifically, it has been shown that in tissue regeneration, committed epithelial cells can transiently reacquire multipotency, therefore contributing to the repair of the tissue [15–20]. According to these findings, an alternative reasoning suggests that loss of specific tumor suppressors or overactivation of certain oncogenes, combined with environmental remodeling, can cause dedifferentiation of committed progenitors and the reacquisition of SC features, therefore increasing the number of potential COs [21–25]. Importantly, the downregulation of lineage-specific genes, in favor of reacquisition of SC-like traits, could represent an essential step to tumor initiation, which may foster CSC formation and maintenance [26]. We can conclude that, although the two concepts of CO and CSC have been often used indiscriminately, it is pivotal to take into account precise definitions in order to better define the molecular mechanisms that drive tumorigenesis and cancer plasticity and improve the development of new targeted strategies.

## 3. Intrinsic and Extrinsic Factors Regulating CSC Function and Self-Renewing

CSCs rely on a number of signaling pathways which control adult SC self-renewal and have key roles in embryonic development and differentiation. Indeed, alteration of stemness signaling, such as NOTCH, SHH, and WNT, can be determinant for tumor initiation and progression. Specifically, aberrant functioning of these pathways in adult SCs may cause uncontrolled cell proliferation and aberrant differentiation, leading to tumorigenesis in a tissue-specific manner [27]. On the other hand, their reactivation in committed cells can favor the induction of reprogramming mechanisms, which lead to the onset of a CSC phenotype. Alteration of stemness signaling can be the result of oncogenic somatic mutations which cause their ligand-independent reactivation. In a physiological setting, NOTCH signaling depends on the communication between contiguous cells and is activated after interaction between a transmembrane ligand and its cognate receptor [28]. During tumorigenesis, ligand-independent activation of the NOTCH pathway occurs through multiple mechanisms, including mutations and complex chromosomal rearrangements, leading to altered stem cell features [29, 30]. In the SHH signaling, binding of SHH ligands to their transmembrane receptor Patched (PTCH) interrupts PTCH-mediated inhibition of Smoothened (SMO) [28]. Active SMO drives a signaling cascade that results in the activation and nuclear translocation of GLI transcription factors (TFs), responsible for SHH target gene expression. Mutations in key members of the pathway which result in both a ligand-dependent and ligand-independent aberrant signaling have been observed in many cancer types. In medulloblastoma, a somatic inactivating mutation of SUFU, which act as a negative regulator of GLI TFs [31], causes an aberrant activation of the SHH signaling [32]. The canonical WNT pathway is among the best characterized stemness signaling, and there are many data supporting its involvement in CSC formation and maintenance [33, 34]. In the presence of active signaling, *β*-catenin translocates to the nucleus, where it interacts with TCF-LEF TFs to transactivate its targets. In the absence of WNT ligands, a multiprotein destruction complex, composed by AXIN, APC, and GSK3*β*, binds cytoplasmic *β*-catenin and mediates its proteasomal degradation [28]. Inactivating APC mutations causes constitutive activation of the pathway and consequent formation of stable *β*-catenin-TCF4 complexes, which drives colon cancer tumorigenesis [35, 36].

CSC identity is also regulated by a number of extracellular factors deriving from the tumor microenvironment (TME). Among the factors of the TME, a variety of infiltrating immune cells, as well as their derived factors, support intratumor heterogeneity [37, 38]. Moreover, extracellular signals such as factors released following tissue damage or inflammation combined with factors coming from the SC niche are important for CSC formation and maintenance, independently from their CO [23]. Chronic inflammation is a hallmark of many cancers, and many evidences indicate that inflammatory signals can regulate tumor initiation, progression, and CSC identity. This is well exemplified in pancreatic cancer in which a cooperative action of inflammatory signals and oncogenic insults targeting progenitor cells induces the formation of CSCs [39]. In the adult pancreas, putative DCLK1^+^ progenitor cells, which are quiescent cells localized in the ducts, play a prominent role in tissue regeneration after injury. Expression of mutant RAS in DCLK1^+^ cells is not sufficient to cause their transformation, but experimental induction of pancreatitis reprograms RAS mutant DCLK1^+^ cells in CSCs able to rapidly produce overt pancreatic cancer.

Altered release of factors from the SC niche can influence cells located outside the niche, which normally are not interested by such signaling. This can induce changes in cell identity, enabling cell transformation and tumor initiation. In the intestinal mucosa, for example, cell identity is determined by the position which a cell occupies along the niche-villus vertical axis, in which the WNT and BMP pathways form a polarized expression gradient [40] (Hardwick et al., 2004). Namely, progenitor and intestinal SC identity and proliferation are defined by high levels of WNT and low levels of BMP in the lower part of the crypt, near the SC niche. On the contrary, cell differentiation is determined by low WNT and high BMP, a condition which characterizes the luminal surface. These gradients are maintained by differential expression of both ligands and antagonist proteins. Specifically, the BMP antagonist GREM1 is expressed in intestinal subepithelial myofibroblasts and in connective tissue SCs and acts at the level of the crypt SC niche (Jaeger et al., 2012; Worthley et al., 2015). However, in hereditary mixed polyposis syndrome (HMPS), a 40 kb genetic duplication causes *GREM1* ectopic expression, with consequent destruction of the BMP gradient along the intestinal epithelium [40]. The resulting alteration of cell fate determination induces cells localized outside the crypt to acquire progenitor-like features, leading to ectopic crypt formation.

**Figure 1:**
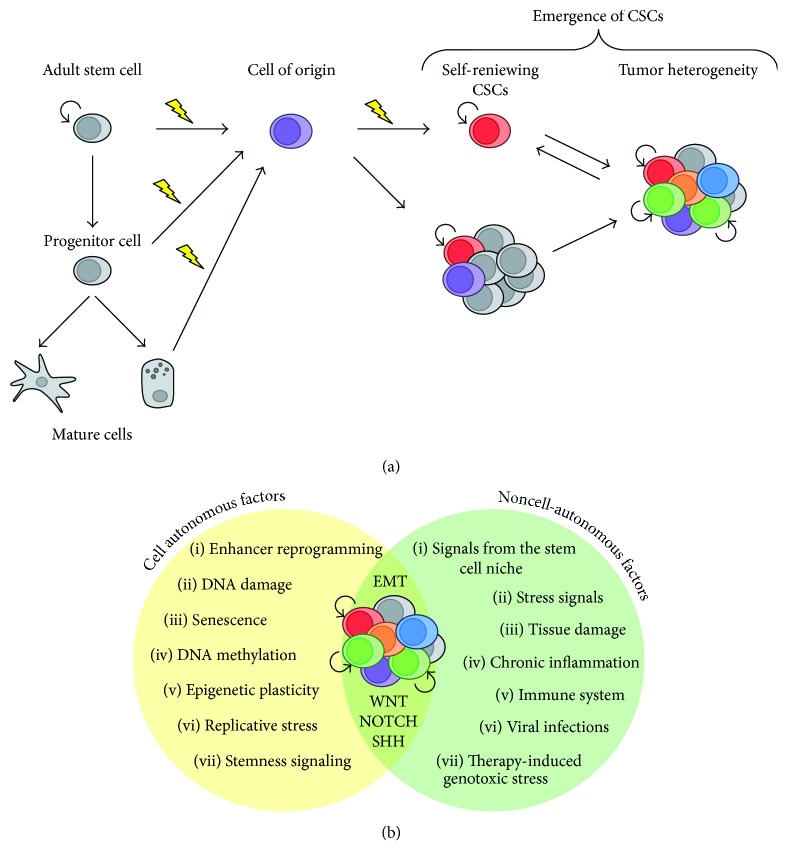
Cellular origin and mechanisms dictating the emergence of CSCs. (a) Cells with different positions in the tissue hierarchy can serve as the cell of origin (CO). Combination of epigenetic and genetic alterations can drive cell reprogramming and the direct onset of a CSC phenotype. In an alternative process, the CO can initiate tumorigenesis, without acquiring stem cell features, and acquisition of further oncogenic aberrations in a cell of the neoplastic progeny may drive CSC formation. CSCs are uniquely responsible for tumor progression and maintenance. (b) A combination of cell autonomous and noncell autonomous factors influence CSC formation and tumor development. Oncogenic mutation of stemness signaling can cause their hyperactivation independently from the tumor microenvironment (TME). Altered signaling from the TME, such as chronic inflammation, or signals from the stem cell niche can induce tumorigenic cell reprogramming. Different signaling converge to regulate epithelial-to-mesenchymal transition (EMT) in tumor initiation and progression.

Increasing evidences indicate that the interplay between different signaling pathways influences self-renewal and proliferation capacity, as well as tumor progression. A study of Schwitalla et al. shows how intestinal tumorigenesis can be driven by an interplay between NF*κ*B signaling and the WNT pathway [41]. Constitutive *β*-catenin activation in intestinal epithelial cells (IECs) induces loss of differentiated cells and massive expansion of crypt SCs, characterized by NF*κ*B activation. Conversely, NF*κ*B modulates WNT signaling, by interacting with *β*-catenin and modulating its DNA binding activity. Accordingly, elevated NF*κ*B signaling in IECs enhances WNT signaling and causes a tumorigenic cell reprogramming process, which leads to the onset of SC-like cells with tumor initiation capacity. One of the most important processes associated with cell reprogramming in both physiological and pathological conditions is the epithelial-to-mesenchymal transition (EMT), namely, a cellular process occurring when an epithelial cell loses adhesion with neighboring cells and acquires mesenchymal properties, such as migration capacity [42]. Many stemness signaling, including TGF*β*, WNT, NOTCH, and SHH can cooperate to induce full EMT responses in cancer [43]. Moreover, both EMT and CSCs rely on the expression of master regulators including SLUG, SNAIL, TWIST1, ZEB1/2, and HIF factors. To this regard, an early study of Guo et al. demonstrated that forced expression of the EMT-TF SLUG with SOX9 in breast cancer cell lines regulates the CSC content and tumorigenic capacity [44]. Moreover, coexpression of SLUG and SOX9 in nonmetastatic breast cancer cells induces EMT and macrometastasis formation. Overall, these data support the notion that CSCs rely on a strict interconnection between SC-related autonomous and nonautonomous factors, whose altered functioning can influence the identity of the CO and induce a committed cell to reprogram to a SC-like state (Figure 1(b)).

## 4. Epigenetic Mechanisms Favoring the Acquisition of Stem-Like Features and the Emergence of CSCs

Epigenetic “modifiers,” which are the factors that directly affect the chromatin features, are among the most abundantly driver-mutated genes in both hematopoietic and solid tumors [45–47]. Indeed, increasing evidences demonstrate that alteration of the epigenetic machinery may favor the formation and the maintenance of CSCs [48–50]. Regardless of the cell which they derive from, the formation of CSCs implies the acquisition of a stem-like transcriptional program, which comprises activation of TF networks and signaling pathways supporting self-renewal and pluripotency [51–55]. To this end, in the early steps of tumor initiation, the epigenetic barriers which determine cell identity must be overcome. Among these, DNA methylation is introduced by DNA methyltransferases (DNMTs) and actively removed by ten-eleven translocation proteins (TETs). Physiologically, DNA methylation occurs mainly at CpG islands (CGI), within promoter regions, mediating their transcriptional repression. In addition, it affects transcriptional elongation, splicing, and genomic stability, by decorating CpG-poor gene bodies and repeat-rich intergenic regions [56]. In many cancers, the promoters of tumor suppressor genes are frequently hypermethylated, giving rise to the so-called “CpG island methylator phenotype” (CIMP). On the contrary, intergenic regions are globally hypomethylated, favoring oncogene transcription and genomic instability [57].

In many cancers, deregulated DNA methylation leads to loss of imprinted monoallelic gene regulation, known as loss of imprinting (LOI) [58]. Data produced in mouse models demonstrate that global LOI events alone promote the onset of cancer, indicating a role for deregulated DNA methylation in inducing tumor-initiating cells [59]. In addition, two recent studies implicate global DNA methylome alterations in hematopoietic tumor disruption. In leukemia, loss or aberrant activity of DNMT3A, in combination with another single oncogenic mutation, induces leukemic SCs (LSCs) and initiates tumorigenesis [60, 61]. Methylation levels depending on DNMT1, instead, have been demonstrated to be functional for the maintenance of the lung, colon, and LSCs and to regulate their tumorigenic potential *in vivo* [62–64]. At the molecular level, it has been demonstrated in multiple cancer types that local hypermethylation at key tumor suppressor genes may predispose the correct oncogenic setting in premalignant cells, which ultimately leads to cell transformation and CSC induction [65–68]. Nonetheless, DNA methylation profiling from gastric, colon, and breast cancers indicates that dysregulation of DNA methylation patterns on both tumor suppressors and oncogenes represents a functional step for the emergence of CSCs [69–71].

Histone posttranslational modifications (e.g., methylation, acetylation, ubiquitination, phosphorylation, and SUMOylation) decorate nucleosomes in the chromatin, defining functional genomics regions, such as promoters, enhancers, and insulators, which ultimately delineate the cellular transcriptional program. Moreover, they also serve as binding platforms for epigenetic “readers,” which are part of multiprotein complexes regulating chromatin structure, genome maintenance, transcription, and replication [72]. Consequently, alterations of the histone modification landscape are widely associate to cancer diseases [45, 46, 57, 73].

Beside DNA methylation, global alteration of histone modification landscapes has been associated to the induction and maintenance of CSCs. Of importance, multiple histone “modifiers” may participate to shape these histone patterns. This concept is well exemplified by the role of enhancer of zeste homolog 2 (EZH2). EZH2 is the catalytic subunit of the polycomb repressive complex 2 (PRC2), which mediates transcriptional repression by introducing trimethylation on lysine 27 of histone 3 (H3K27me3) [74]. In both breast cancer and large B cell lymphomas, EZH2 is hyperactivated and it has been demonstrated to be sufficient to transform human mammary epithelial cells and required for early steps of lymphomagenesis, indicating that these tumors rely on increased H3K27me3 [75–77]. Similarly, in prostate cancer, EZH2-mediated H3K27me3 mediates gene silencing of tumor suppressors [78]. On the contrary, in pediatric GBMs, the K27M mutation of histone variants H3.1 and H3.3 leads to reduced activity of EZH2, subsequent genome-wide reduction of H3K27me3, and reprogramming towards a more primitive stem-like state [79, 80]. Accordingly, in myeloid malignancies, loss of EZH2 function is sufficient to induce a self-renewal-supporting transcriptional program and leukemogenesis [81, 82]. These evidences indicate that deregulation of the H3K27me3 landscape, hence the transcriptional repression, is the driving force for the emergence of CSCs, independently of the originating mutation of EZH2.

Perhaps, the best documented example of a histone “modifier” involved in induction of LSCs is represented by the mixed lineage leukemia (MLL) histone methyltransferase [83]. Generally, in different types of leukemia, chromosomal rearrangements lead to MLL oncogenic fusion proteins (i.e., MLL-AF9 and MLL-ENL), which lack the catalytic domain but are able to reprogram committed cells (both hematopoietic SCs and myeloid progenitors) towards LSCs and initiate tumorigenesis [84–86]. Nonetheless, it is reported that MLL oncogenic fusions may also require the repressive activity of polycomb repressive complex 1 (PRC1), which monoubiquitinates histone H2A on lysine 119 (H2AK119Ub1) and acts in concert with PRC2 to mediate transcriptional repression [74]. In particular, the BMI1 subunit of PRC1 is necessary to mediate repression of tumor suppressors in myeloid progenitors, thus favoring the reprogramming towards a stem-like program in LSCs [87, 88]. In agreement, BMI1 is also required to represses tumor suppressor genes and initiate CSC self-renewal in solid tumors [89, 90].

A further layer of the epigenetic landscape is represented by the nucleosome structure, dynamics, and density, controlled by ATP-dependent chromatin remodeling complexes which move, eject, or restructure nucleosomes. Four families of nucleosome remodelers are present in eukaryotes, SWI/SNF, ISWI, CHD, and INO80, which differ for their functional activity, protein domains, and subunits [91]. Interestingly, the SWI/SNF complex is frequently implicated in malignant transformation, with more than 20% of human cancers carrying mutations in its subunits [92]. Functional evidence for a role of SWI/SNF complex in inducing CSC is reported for pediatric rhabdoid tumors. Indeed, loss of SMARCB1, a subunit of the complex, is the unique genetic alteration which drives rhabdoid tumors and is associated with blocking of the differentiation, reprogramming towards an oncogenic transcriptional program and activation of tumorigenic signaling [93–95]. Similarly, ARID1A, another subunit of SWI/SNF, acts as tumor suppressor in colon cancer and its loss is solely able to activate an oncogenic transcriptional program and promote invasive colon adenocarcinomas in mouse [96].

Collectively, these data strongly indicate that deregulation of the DNA methylation and histone modification landscapes represents a key step for the onset of CSCs. Even though in different cancer types, the epigenetic profile of CSCs may be altered by different lesions, CSCs possess a more accessible, plastic, and hyperdynamic chromatin with respect to their differentiated counterparts [97]. Overall, it is coming evident that epigenetic alteration can play an important role in CSC formation and maintenance and future studies should highlight the molecular insights governing CSC plasticity.

## 5. Global Epigenetic Landscape Reshaping and Enhancer Reprogramming in the Emergence of CSCs

Apart from identifying the single aberrations of chromatin “modifiers,” applying next-generation sequencing technologies to profile the epigenome of cancer cells permits to unveil global epigenetic rearrangements occurring during tumor progression. Cells possess a highly organized chromatin conformation, which is largely reshaped upon malignant transformation [73, 98, 99]. Physical constrains, such as insulators and topological associated domains (TADs), limit and affect the activity of transcriptional regulatory elements, while lamina-associated domains (LADs) mainly colocalize with large organized chromatin modifications (LOCKs) and define wide heterochromatic regions of silenced genes in committed cells [100, 101]. Alterations of these higher order macrodomains have been functionally linked to tumorigenesis [102–106]. Of importance, alteration of chromatin topology impinges on functional regulatory elements such as enhancers, which comprise arrays of TF binding motifs and boost transcription of related promoters over long genomic distances [107]. Enhancers are invariantly decorated with monomethylation of lysine 4 on histone 3 (H3K4me1) and with acetylation of lysine 27 on histone 3 (H3K27ac), in their active form. Moreover, enhancers are enriched for the binding of chromatin factors such as p300/CBP, the main histone acetyltransferases which mediate H3K27ac, and mediator, a long-range interaction facilitator [107]. Given those features, it is evident that aberrant activity of various chromatin “modifiers” can affect the enhancer integrity and functionality, opening a window of opportunity for the cell to reprogram its transcriptional landscape and finally its own identity. Accordingly, enhancer malfunction is gathering importance in the onset of cancer, as it may favor the acquisition of a stem-like program in committed cells [108, 109]. Nonetheless, only few examples are available to date, which clearly correlate enhancer reprogramming and the emergence of CSCs [96].

**Figure 2:**
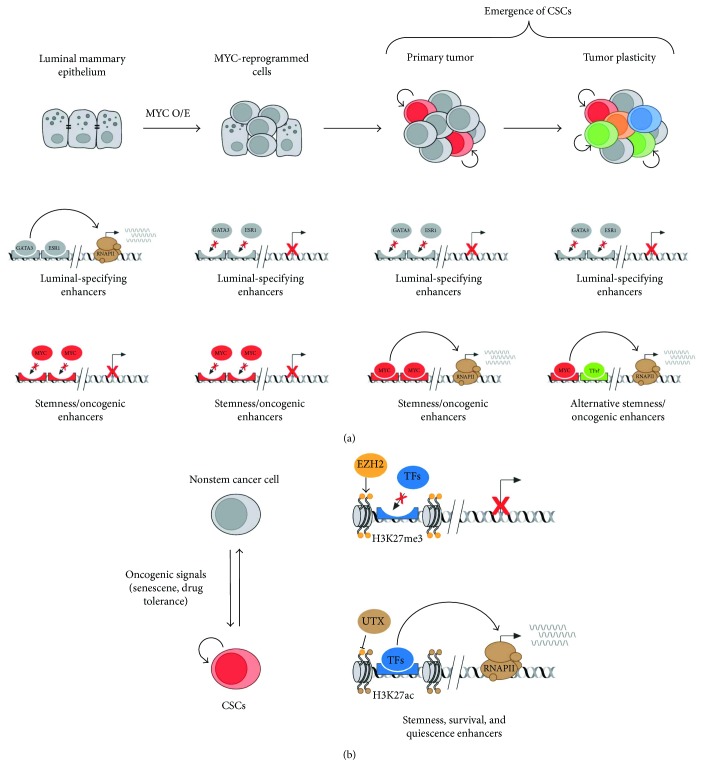
Enhancer reprogramming in the induction of CSCs and tumor plasticity. (a) Luminal mammary epithelial cell identity is specified by a transcriptional program regulated by activation of enhancers bound by luminal-determining transcription factors, such as GATA3 and ESR1. Upon overexpression of MYC, these enhancers are turned off, due to the transcriptional repression of GATA3 and ESR1. Consequently, MYC binding on de novo enhancers leads to activation of oncogenes and stemness genes. This enhancer reprogramming favors tumorigenesis and the formation of basal-like primary breast cancers in mice. Further activation of alternative enhancers regulating stemness and oncogenesis by yet unknown transcription factors may favor the emergence of new CSCs and tumor plasticity. (b) In glioblastoma, oncogenic signals, such as senescence and therapy-induced genotoxic stress, mediate the conversion between nonstem cancer cells and CSCs. This mechanism of tumor plasticity is governed by a global enhancer reprogramming: distal elements regulating stemness, survival, and quiescence shuttle between a repressed state, marked by H3K27me3, and an active state, marked by H3K27ac, which is imposed by the alternative activities of EZH2 and UTX.

Interestingly, we recently provide additional data demonstrating the role of enhancer reprogramming during the early steps of tumorigenesis in an *in vitro* and *in vivo* model of breast cancer [26] (Figure 2(a)). We show that human luminal mammary epithelial cells (IMEC) lose their cell identity upon overexpression of the known pluripotent and oncogenic TF MYC [110, 111]. This is due to the MYC-mediated transcriptional downregulation of luminal-specific TFs, leading to the decommissioning of the enhancers which dictated the luminal mammary epithelial identity. In addition, IMEC overexpressing MYC are able to grow as mammospheres and acquire stem-like features, such as self-renewal and multilineage differentiation capacity. This is paralleled by the reprogramming towards a progenitor/SC-like transcriptional program, which is achieved by the activation of de novo enhancers. Indeed, mammosphere-specific distal regulatory enhancers drive the expression of both TFs and signaling pathways, usually activated in adult and cancer SCs [26]. We demonstrated that the enhancer reprogramming occurring *in vitro* is also maintained in the *in vivo* setting, indicating an oncogenic function for the de novo enhancers and a putative role in the emergence of CSCs, which sustain the breast cancer growth in mice [26].

Even though enhancer reprogramming has been functionally linked to the formation and maintenance of CSCs, further research is required to causally link activation of oncogenic enhancers and malignant transformation. Following enhancer activity during tumorigenesis, by the means of reporter tools, and turning them off with genetic engineering *in vivo*, will definitely clarify their impact on tumor progression.

## 6. Cell Reprogramming in Tumor Initiation

The limited knowledge regarding the effect of oncogenic aberrations on different committed cells represents a major issue for cancer biology. Indeed, alternative tumor reprogramming processes may differentially impinge on cancer heterogeneity and progression. Two recent publications greatly contributed to elucidate concerns regarding the CO of breast cancers, as well as the effect of oncogenic insults in specific cell lineages on tumor heterogeneity and clinical outcome [112, 113]. Of note, the mammary gland is a hierarchically organized organ, constituted by an inner layer of luminal cells (LCs) surrounded by an outer layer of basal cells (BCs) [114]. Using a genetic lineage-tracing approach in mice, Van Keymeulen et al. express a mutant isoform of PIK3CA in the BC subtype. Interestingly, this causes the formations of tumors with a luminal phenotype. On the other hand, tumors originated from mutant PIK3CA expression in LCs include both luminal and aggressive basal-like tumors. Moreover, while expression of oncogenic PIK3CA in luminal progenitors causes a multilineage differentiation at the early stage of tumor initiation, its expression in unipotent BCs gives rise to functional LCs. The molecular mechanisms upon which such oncogene-induced reprogramming relies are common and cell lineage-specific and are influenced by both the CO first interested by the oncogenic heat and the cell lineage in which transformed cells evolve. Similar observations are made in the study of Koren et al., which reveals that the heterogeneous phenotype characteristic of mammary tumors driven by constitutive PI3K signaling is caused by reprogramming of lineage-restricted COs to a multipotent state from which cells further differentiate. Moreover, they show that tumor aggressive behavior is strictly dependent on the identity of the CO: expression of mutant PIK3CA in basal cells mainly give rise to benign tumors, whereas its expression in luminal cells mostly coincides with aggressive tumors. Altogether, these studies show that a single oncogenic insult in committed cells can cause dedifferentiation to a multipotent SC-like state, therefore suggesting that this mechanism could contribute to tumor heterogeneity. Moreover, the significant similarity between these mouse models and human breast cancer gene expression profiles indicates that it is plausible that similar mechanisms may concern human mammary gland tumorigenesis. In a recent work, Ye et al. use a similar approach to determine the relative contribution of EMT-TFs to CSC formation, during multistep mammary gland tumorigenesis [115]. Using genetically engineered knock-in reporter mice, combined with an organoid culture system, they observe that, while SLUG TF is expressed in cells of the basal compartment and is involved in the determination of the normal mammary SC state, tumor initiation is driven by SNAIL expression. Specifically, SNAIL is expressed in luminal cells and drives acquisition of a basal-like phenotype and CSC properties, through induction of EMT. Of note, these observations are in line with the above-described findings and corroborate the hypothesis that CSCs can originate from cells different than adult SCs and can exploit different molecular mechanisms to activate the signaling necessary for their maintenance. Of importance, similar mechanisms have been observed also in other models of tumorigenesis. Melanoma is a cancer generated by transformation of the melanocyte lineage, which originate from the neural crest [116]. The work of Kaufman et al. proves that melanoma initiation is driven by reactivation of a neural crest progenitor program in pretumorigenic melanocytes [117]. Notably, during development, *crestin* is expressed in neural crest stem and progenitor cells, but it gets specifically reactivated at the early stage of tumorigenesis, as shown by performing live imaging of transgenic zebrafish *crestin* reporters. Of importance, Crestin^+^ cells derived from melanoma tumors are characterized by expression of a neural crest-specific gene signature including SOX10, DLX2, and DFAP2. Altogether, the reported cases indicate that cell reprogramming processes could be a relevant mechanism involved in tumorigenesis.

## 7. Cancer Cell Plasticity: Cell Reprogramming Processes in Tumor Maintenance

There are several evidences indicating not only that not all cancers respect a rigid hierarchical organization but that CSC and non-CSC populations are flexible and can interchange, in response to environmental cues. This notion can be clearly exemplified by two recent studies in which researchers investigate whether loss of intestinal CSCs can be compensated by the remaining differentiated cancer cells [118, 119]. Using cells from genetically engineered mice, which express the inducible suicide gene diphtheria toxin receptor (DTR) in LGR5^+^ SCs, de Sousa e Melo et al. establish *in vitro* organoid cultures in which they apply genome editing techniques to introduce several mutations necessary to drive colorectal cancer onset. After transplantation in mice, the organoids form tumors. Interestingly, following selective depletion of LGR5^+^ CSCs, the tumors do not regress but remain at a constant size. In this phase, tumors are maintained by proliferating LGR5^−^ cells, which compensate the ablation of the LGR5^+^ CSC pool, by upregulating MYC target genes, responsible for cell-cycle progression. Moreover, after suspension of diphtheria toxin treatment, LGR5^+^ cells rapidly reappear and tumor starts regrowing, supporting a model in which various types of LGR5^−^ cancer cells can dedifferentiate and replenish the LGR5^+^ CSC pool. In the study by Shimokawa et al., the dynamics of LGR5^+^ cell-driven colorectal cancer is investigated by generating organoids in which LGR5^+^ cells can be selectively killed by inducing the suicide gene caspase 9. Ablation of LGR5^+^ cells causes a significant reduction in tumors size, which is recovered thanks to reemergence of LGR5^+^ CSCs after stopping the treatment. Also in this case, an initial compensatory proliferation of remaining LGR5^−^/KRT20^+^ cells is observed, but it is insufficient to ensure tumor volume maintenance. To verify whether tumor recovery is effectively driven by newly formed LGR5^+^ cells derived from differentiated LGR5^−^/KRT20^+^ cells, Shimokawa et al. generate KRT20-CreER knock-in lines of organoids to follow KRT20^+^ cell fate *in vivo*. Interestingly, postmitotic LGR5^−^/KRT20^+^ cells were able to reenter cell cycle, generating large clonal colonies containing newly formed LGR5^+^ cells.

These studies suggest that, in response to specific environmental pressures, differentiated cancer cells can be stimulated to reprogram, therefore recovering proliferation capacity and CSC-like features, ensuring tumor maintenance and progression. This suggests that specific signals coming from the TME are sensed by cancer cells of the tumor bulk and fuel cancer cell plasticity, causing switch from a temporary “static” hierarchical condition to a reprogrammed state. In this context, the influence that the CSC niche has on tumor bulk must be taken into account. It is also important to take in consideration the fact that cancers are evolving entities and that further genetic and epigenetic insults can be added to the tumoral landscape during tumor progression, fueling CSC plasticity. Finally, another possible scenario is that cancer plasticity represents a “default” dominant tumor feature, not necessarily activated by the incoming of new specific stimuli or new oncogenic insults. In this case, cell reprogramming processes would be mainly stochastic events, which happen over tumor evolution and that represent an advantageous feature in case of damage at the CSC population.

## 8. Reversible Epigenetic States Support Cancer Cell Plasticity

Reversible epigenetic alterations play a central role in cancer cell plasticity, favoring or counteracting the activation and maintenance of the SC-like transcriptional program that sustains tumor progression. Similarly, to cell reprogramming towards induced pluripotent cells, different epigenetic regulators participate in stabilizing SC-like epigenetic states, whereas others are involved in modulating cancer cell plasticity. Considering that the interconversion between cellular states depends on the capacity to switch off a cell-specific transcriptional program while activating SC-related genes, it is not surprising that chromatin regulators playing a role in maintenance of cell identity are frequently involved in cancer cell plasticity. For example, altered expression of key components of polycomb repressive complexes (PcGs) and members of the trithorax group (TrxG) is correlated with sustained stemness of CSCs in different tumors [120–122]. In GBM, several PcG and TrxG components play a critical role in the maintenance of CSCs and in cancer cell plasticity [123–127]. In adult GBM patients, the SET protein MLL5 that is overexpressed in CSCs downregulates the expression of H3.3, causing local reorganization of chromatin structure [124]. Of importance, forced expression of MLL5 in nonstem cancer cells is sufficient to induce cell plasticity by repressing proneural differentiation, thereby eliciting a SC state. Similarly, reduced expression of H3.3 favors self-renewal properties, phenocopying the effect of H3.3 mutations in pediatric GBM [80]. This work highlighted the key role of reversible chromatin structures in establishing functional properties of CSCs. On the same line, it has been recently established that the linker histone variant H1.0 is expressed at low levels in CSCs, permitting the establishment of a SC-specific epigenetic state that facilitates the expression of oncogenic and self-renewal genes. Importantly, perturbing H1.0 protein level directly affects self-renewal capacity, promoting differentiation in nonstem cancer cells, thus hampering their tumorigenic capacity *in vivo* [128]. It is plausible that, through yet undefined regulatory mechanisms, these chromatin-associated proteins could indeed influence the responsiveness of cancer cells, increasing their adaptability to intrinsic and extrinsic cues, thus enhancing cancer cell plasticity. Further studies will permit to elucidate the contribution of chromatin components and nuclear architecture to cancer cell plasticity.

Cancer is a chronic and heterogeneous disease, which causes persistent tissue damages and local inflammatory responses. This harmful environment is causative of cellular senescence that represents a first barrier towards cancer progression, by counteracting the expansion of otherwise damaged preneoplastic cells [129]. Different oncogenic-associated features induce senescence including replicative stress, oncogene overactivation, telomere shortening, sustained DNA damage, therapy-induced genotoxic stresses, and a proinflammatory microenvironment. Of importance, cancer-associated senescence elicits a protumorigenic milieu through the production of secretory factors, including extracellular proteases, cytokines, and chemokines, named senescence-associated secretory phenotype (SASP). Recent findings demonstrated that *in vivo* cell reprogramming towards a pluripotent SC state is supported by tissue damage and cellular senescence, with SASP playing an instructive noncell autonomous function [130]. More importantly, Milanovic and collaborators investigated whether therapy-induced senescence (TIS) promotes cancer stemness and plasticity [131]. In this work, it has been shown that transient TIS induces the activation of a SC-like program, which supports the formation and plasticity of CSCs *in vivo*.

Cancer cell plasticity is counteracted by those epigenetic barriers that have been previously shown to hamper *in vitro* cell reprogramming, such as Suv39h1-mediated H3K9me2/3 deposition [131, 132]. New insights on those epigenetic barriers were recently reached by analyzing the chromatin state of adult and CSCs, in response to tissue damage [133]. While resident SCs activate a transient stress-induced epigenetic and transcriptional program, CSCs overactivate stress-responsive enhancers, therefore overriding cell lineage specification. It is plausible that tumor microenvironment resembles a chronic damaged tissue, favoring the activation of stress-responsive enhancers, which support CSC plasticity [133].

Many reports have now shown that epigenetic plasticity enables cancer cells to adapt to exogenous stresses, including conventional and targeted therapies. Different nonmutational drug resistance mechanisms are involved in supporting the formation and maintenance of residual cancer “persister” cells [134–136]. Among others, the functional role of enhancer rewiring, in response to genotoxic stresses, is upcoming a relevant and common mechanism to drive drug-induced transcriptional adaptation and tumor relapse [137]. For example, adaptive chromatin remodeling at enhancers drives glioblastoma CSC (GSC) plasticity and supports drug tolerance [135]. Specifically, GSCs can reversibly transit to a slow-cycling state in response to targeted kinase inhibitors [135]. Of importance, GSC presisters are characterized by the reactivation of primitive developmental programs through the upregulation of H3K27me3 demethylase KDM6A/B, thus facilitating the activation of *cis*-regulatory elements required for drug tolerance (Figure 2(b)). Further investigations, focusing on the relationship between stress-induced signals and epigenetic reprogramming of enhancers in CSCs, are warranted to determine their contribution to tumor reprogramming and cancer progression.

## 9. CSC Resistance to Therapy

Current failure with cancer treatment is not usually due to a lack of primary response, but to tumor recurrence after therapy. It is widely accepted that CSCs are closely related to pathological features which result in worse clinical prognosis. Most of all, CSCs show higher resistance to chemotherapy and radiotherapy than other cancer cells and can therefore escape from the conventional cytotoxic treatments, driving tumor relapse [138, 139]. Of note, medical treatment can result in the enrichment of the CSC pool, suggesting an induction or positive selection for cancer cells with SC properties [140].

Resistance to standard antiproliferative chemotherapy and radiation was initially considered an intrinsic feature of normal SCs and CSCs, acquired through multiple independent mechanisms, such as high expression of drug efflux pumps and relative resistance to oxidative stress or DNA damage [141]. In comparison to their differentiated progeny, CSCs show a superior and more efficient DNA damage response (DDR) [142]. This concept is well exemplified by GBM, where patient-derived CSCs exhibit increased activity of the DDR targets with consequent rapid and enhanced DNA repair response following irradiation [143]. Moreover, ovarian CSCs are characterized by enhanced expression of DNA polymerase eta, which ensures high tolerance to DNA damage [144].

Of importance, the ability of CSCs to assume a quiescent condition has also shown to influence therapy resistance [134]. For example, one study performed in mouse squamous cell carcinoma indicated that perivascular TGF-*β* causes heterogeneous signaling at tumor-stroma interface and confers quiescent properties to neighboring CSCs [145]. Interestingly, TGF-*β*-responding CSCs show resistance to anticancer therapies, therefore driving tumor recurrence. However, different tumorigenic settings showed that slow-cycling LGR5^−^ SCs resulted highly radiosensitive [146], suggesting that multiple, yet poorly defined factors can influence CSC therapy responsiveness. In addition to these endogenous features, numerous evidences indicate that also the surrounding TME contributes to CSC-related resistance to therapy, by directly regulating their physiology. For example, it has been observed that DNA damage to components of TME promotes the secretion of growth-promoting factors which enable cancer cells to survive cytotoxicity, therefore promoting therapeutic resistance [147].

A further critical factor that must be taken into account in the context of drug resistance is CSC plasticity. There are several observations corroborating the fact that CSCs can generate clones of cancer cells carrying different combinations of driver mutations, thereby increasing the chances to develop resistance to anticancer therapy [148]. Nevertheless, as discussed above, many recent findings show that the reverse mechanism is also possible therefore replacing lost CSCs through plasticity. Considering that the main flaw of current medical therapies consists in the fact that they target actively dividing cells, it is plausible that quiescent differentiated cancer cells could represent a further source of resistant. In this view, not cycling cancer cells could survive therapy, therefore having the possibility to further refill the CSC pool after dedifferentiation and contribute to tumor relapse.

## 10. Perspectives and Concluding Remarks

Since it has been demonstrated that cells with CSC properties are drivers of tumor initiation and maintenance, elaboration of therapies aimed at their elimination is considered to be essential [42]. Several studies aimed at depleting CSC populations by targeting their surface markers, through use of antibody-drug conjugates (ADCs). To this purpose, experiments performed in xenograft and in genetically engineered mouse models of intestinal tumorigenesis succeeded in eliminating LGR5^+^ CSCs, demonstrating potent antitumor efficacy [149, 150]. In high-grade pulmonary neuroendocrine tumors, there are evidences indicating that use of an ADC targeting NOTCH ligand DLL3 effectively eradicates CSCs [151]. However, many pitfalls arise also from the cancer cell plasticity paradigm which implies that nonstem cancer cells can replenish the CSC counterpart, in response to targeted therapy. By reasoning in this perspective, an alternative strategy could be to block oncogenic signals that are indispensable to acquire, maintain, or reverse to cancer stemness. In a xenograft model of colon cancer, in which WNT pathway hyperactivation is caused by fusion events between *RSPO3* and *PTPRK* genes, treatment with anti-RSPO3 function-blocking antibody causes a rapid loss of CSC function and consequent reduction of tumorigenicity and increment of cell differentiation [152]. Although there is clinical relevance, these kinds of approaches are weakened by the many possible toxic side effects caused by depletion of the adult SCs relying on the same pathways or expressing the same surface molecules [28, 153–156]. Moreover, despite the encouraging results, these strategies have many disadvantages and limitations due to the lack of CSC-specific markers and the intratumor heterogeneity in their expression, as well as the fact that not all the CSCs present in a tumor may rely on the same stemness signaling. For these reasons, more specific therapies must be conceived, which could limit the window of the targeted population. Another challenging factor to take in consideration is that with tumor progression, CSC population may evolve, thus escaping to the therapeutic treatment [157].

An alternative CSC-related feature that could be exploited is their ability to acquire a quiescent behavior. A study performed in bladder cancer revealed that, in a mechanism similar to that driven by normal SCs during wound repair, chemotherapy-induced damage stimulates quiescent CSCs to proliferate [158]. Interestingly, this is caused by prostaglandin E_2_ (PGE_2_) released by apoptotic cancer cells, a factor known to stimulate SC expansion. Notably, use of a PGE_2_-neutralizing antibody, as well as block of PGE_2_ signaling, through the use of a pharmacological inhibitor of the enzyme mediating PGE_2_ production, COX2, is sufficient to abrogate CSC repopulation. Clearly, this kind of strategy is efficient in preventing therapy-induced CSC awakening but does not eliminate them. A more feasible approach is that of targeting quiescent CSCs basing on their peculiar metabolism, a strategy which allows to discriminate CSCs from cycling cancer cells and that, in combination with other treatments, can contribute to the development of more efficient therapies [159].

Therapies based on the inhibition of multiple epigenetic regulators are also promising, considering the potent effects they have on gene expression that allow to target CSCs in a more specific way. DNMT inhibitors (DNMTi), histone deacetylase inhibitors (HDACi), and inhibitors of the bromodomain and extraterminal motif proteins (iBET), approved by the US Food and Drug Administration (FDA), are actually in clinical trials for several malignancies and they are reviewed in detail in [160]. Of note, advancements made in nonsolid malignancies, until now, are more than those made in the study of solid tumors, due to the still limited knowledge of the epigenetic regulation of CSCs in that kind of diseases and the diffuse challenge caused by the toxic effect that does not spare normal SCs relying on the same epigenetic regulators.

In the context of targeted therapeutics, the use of small molecule inhibitors and monoclonal antibodies targeting kinases showed remarkable antitumor response in many cancers [137]. Notably, kinase gene amplification or mutation represents key oncogenic drivers, due to the pivotal role of kinases in the integration of multiple cellular networks, in response to intracellular and extracellular signals. In spite of the undeniable positive responses observed after treatment with kinase inhibitors, there is a diffuse tendency to develop resistance, over time. Recent approaches are showing the therapeutic value of adopting a combinatorial approach in which besides using targeted therapeutics, epigenetic drugs are simultaneously used to hamper the overcoming drug tolerance. As exemplified by the study of Liau et al., chromatin remodeling enzymes play an essential role in the development of cancer cell-adaptive transcriptional responses to targeted therapies [135]. In glioblastoma CSCs, transition to a quiescent, drug-resistant state, after receptor tyrosine kinase (RTK) inhibition, is dependent on the upregulation of KDM6A/B, which reconfigures H3K27me3 landscapes. Of note, treatment with a small molecule inhibitor is sufficient to restore H3K27me3 levels in quiescent CSCs. These data indicate that the development of combined strategies which include the use of small molecule inhibitors against these epigenetic regulators can be crucial to hindering the transcriptional remodeling effect, responsible for drug resistance. We can postulate that the limited efficacy of available therapeutic options depends on the intrinsic plastic nature of CSCs, which allows them to adapt to cell autonomous and nonautonomous factors. Moreover, current limited knowledge of the mechanisms which drive CSC formation and maintenance further hampers the design of efficacious therapies. For these reasons, future research lines must be finalized to a better understanding of the molecular factors which dictate CSC identity and plasticity, with a particular effort in the development of combinatorial treatments, which may target both oncogenic cell signaling and epifactors.
